# Sexual violence and risk factors among night shift female college students in Hawassa city, South Ethiopia, 2020

**DOI:** 10.1186/s12905-022-02150-w

**Published:** 2023-01-21

**Authors:** Hawi Leul Esayas, Hirut Gemeda, Teshome Melese, Gebremariam Temesgen Birgoda, Bezabih Terefe, Samuel Abebe, Muluken Bekele, Fitsum Wolde, Bamlaku Birie

**Affiliations:** 1 Departments of Midwifery, College of Medicine and Health Sciences, Arba Minch University, Arba Minch, Ethiopia; 2 Department of Midwifery, Hawassa University, Hawassa, Ethiopia; 3 Schools of Public Health, Arba Minch University, Arba Minch, Ethiopia; 4 Department of Midwifery, Mizan Tepi University, Mizan Tepi, Ethiopia

**Keywords:** Sexual violence, Night shift female students, Hawassa city

## Abstract

**Background:**

Sexual violence is any sexual act, attempt to obtain a sexual act, unwanted sexual comments or advances, against a person’s sexuality using coercion, by any person regardless of their relationship to the victim, in any setting. Several studies were undertaken on sexual violence among students in general. Nevertheless, there is paucity of information about sexual violence among night shift students in particular; even if they are more vulnerable to sexual violence due to the nature of the class time. The government has embraced legal and policy frameworks to discourse the problem of sexual violence in Ethiopia; nevertheless, the problem still is quite pervasive.

**Objective:**

This study was aimed to assess the prevalence of sexual violence and risk factors among night shift female college students in Hawassa city.

**Methods:**

Institution-based cross-sectional study design was employed. A structured questionnaire was used to collect the data from 345 study participants. Systematic random sampling technique was used to choice study participants. Epi data version 3.1statistical software and Statistical Package for Social Sciences version 22.0 were used to enter and analysis the data. Both bivariable and multivariable logistic regression analyses were performed to recognize risk factors. *P* values < 0.05 with 95% confidence level were used to state statistical significance.

**Results:**

A total of 330 students were participated in the study making a response rate of 95.6% with a mean age of 24.9. The prevalence of last 12 month sexual violence was 202 (61.2%) 95% CI (55.8, 66.4) which includes rape, attempted rape and sexual harassment. The 12 month prevalence of each form of violence was 46 (13.9%) 95% CI (10.6, 17.9), 23 (6.9%) 95% CI (3.6, 10.9) and 163 (49.4%) 95% CI (46.2, 53.6) of rape, attempted rape and sexual harassment respectively. The independent predictors of sexual violence having a father with no formal education (AOR = 2.39, 95% CI 1.04, 5.33) presence of multiple sexual partners (AOR = 3.44, 95% CI 1.64, 7.2), having sexual partner (AOR = 1.89, 95% CI 1.03, 3.5), and consuming alcohol (3.55, 95% CI 1.84–6.85) by the victims.

**Conclusion:**

This study shown that the prevalence of sexual violence is high among night shift female college students in Hawassa city. Having a father with no formal education, drinking alcohol, having multiple sexual partners and having sexual partner were more likely to have sexual violence within the last 12 month. Thus, students should prevent themselves from health risky behaviors and Hawassa educational bureau should be work on awareness creation concerning women empowerment with in marriage and Further broad and longitudinal studies are needed to determine the predictors of the problem among female students at Hawassa and Ethiopia as a whole.

## Background

One of the world’s most dominant human rights violations is violence against women and girls it taking place every day, many times over, in every corner of the globe. Violence against women and girls is defined as any act of gender-based violence that results in, or is likely to result in, physical, sexual or mental harm or suffering to women and girls, including threats of such acts, coercion or arbitrary deprivation of liberty, whether occurring in public or in private life [1].

One form of interpersonal violence is sexual violence. According to the WHO definition, Sexual violence is any sexual act, attempt to obtain a sexual act, unwanted sexual comments or advances, against a person’s sexuality using coercion, by any person regardless of their relationship to the victim, in any setting [2].

Sexual violence can occur in different forms these include rape with-in marriage, rape by a stranger, sexual abuse of children, forced abortion, forced prostitution, sexual abuse of mentally or physically disabled peoples, forced sterilization, forced exposure to pornography, female genital mutilation, trafficking for purposes of forced prostitution but not limited to this [3]. It takes place within a variety of settings, including the home, workplace, schools, and the community [4].

Schools are an important institution or organization environment that may women’s routinely exposed to sexual violence [5]. Among five college females, one of them are victims of acquaintance rape during their academic career and less than 5% of college women who are victims of sexual assault report their victimization globally [6]. A study done on undergraduate students from Columbia University and Barnard College in New York City, established that sexual assault victimization among college students in the United States was higher [7].

Sexual violence is common; it occurs in every culture, in all levels of society and in every country of the world. WHO multi-country study on violence against women in ten countries reported that the lifetime and previous 12 months prevalence of sexual violence against women ranges between 15–71% and 4–54%, respectively [8].

The study done in Chile college students experiences of rape, attempted rape, and other types of forced sexual contact since age 14 were reported by 12.3%, 10.8%, and 25.1% of subjects, respectively [9]. Sexual violence in developing countries is an acute problem because it is related to HIV infections, especially in countries where the infection rate is high. The magnitude of sexual coercion ranges from 5 to 33% among Ugandan university students [10].

The studies conducted in different parts of our country, Ethiopia on prevalence of sexual violence shows different result. A Study conducted among night time secondary and high school students in Hawassa city reported that lifetime prevalence of sexual violence was found out to be 86.4% [4]. The study done in Bahir Dar town showed that among private college female students, 37.3% of college females have experienced some form of unwanted sexual contact [11]. A study from Nekemte town revealed the lifetime prevalence of completed rape, attempted rape and sexual harassment was 20.8%, 23.1% and 41.3% respectively [12].

The consequences of sexual violence were absenteeism, grade repetition, dropout of school, negative and confused believes of self, exposure to sexually transmitted infections and unwanted pregnancy [13]. Behavioral, lifestyle, and relationship factors have all been identified as risk factors that increase a women’s vulnerability to sexual violence victimization. However, factors like discussion about reproductive issues with family and family control were factors which decrease vulnerability to sexual violence [14].

The government has adopted legal and policy frameworks to address the problem of sexual violence in Ethiopia. Furthermore, institutional structures including specialized units dealing with the problem of sexual violence have been established at different levels of government. But, because the practice of sexual violence is long rooted in cultural, traditional practices, and religious beliefs, Moreover, Ethiopian Ministry of Health has developed a standard operating procedure for the response and prevention of sexual violence in Ethiopia in 2016,while the problem still is quite pervasive [15, 16]. Besides, several studies were undertaken on sexual violence among students in general. Nevertheless, there is paucity of information about sexual violence among night shift students in particular; even if they are more vulnerable to sexual violence due to the nature of the class time. Additionally, the nature of a city; which is the place of many strangers make night shift students susceptible to sexual violence, due to these experts suggested conducting this study. Therefore, this study was aimed to assess the prevalence of sexual violence and its associated factors among night shift female college students in Hawassa city.

## Methods and materials

### Study setting, design and population

Institution based cross sectional study was done in Hawassa city administration which is found in Sidama Region, Ethiopia. It is located 275 km away from Addis Ababa, the center of Ethiopia. The city is one of the fastest-growing cities of Ethiopia which is crossed by one main asphalted road from Addis Ababa to Moyle.

There are four governmental and twenty-eight private colleges in the city. There are different departments in each college: accounting, management, marketing, secretarial science, surveying, industrial electrical machine, information technology etc. each provided be level 1–5, diploma, degree, and some of them up to masters level. There were 2961 night shift students in governmental colleges; from these 777 were females, and also females comprise 1606 out of 2751 private college nights’ students. All night shift female students in Hawassa city private and governmental colleges were the target populations, in which second year and above were considered to draw samples. Selected night shift 2nd year and above female students at the time of the study were participants. All methods were performed in accordance with relevant guideline.

#### Source population

All night shift female students in Hawassa city private and governmental colleges.

#### Study population

All night shift 2nd year and above female students in selected private and governmental colleges.

#### Study participants

Selected night shift 2nd year and above female students who were willing and available during the study period.

### Sample size determination

The Sample size was calculated using a single proportion formula.

$${\text{n}} = \frac{{{\text{Z}}^{2} {\text{p}}\left( {1 - {\text{p}}} \right)}}{{d^{2} }} = \frac{{1.96^{2} \times 0.266\left( {1 - 0.266} \right) }}{{0.05^{2} }} = 300$$

From the study done on female night school students in Hawassa city 26.6% experienced attempted rape in their lifetime [4] p = 0.266, at marginal error of 5% with 95% confidence level which is 1.96.

For possible non-response during the data collection time, 15% added which gave a final sample size of 345.

### Sampling procedures

There are two governmental and twenty-two private colleges which have been providing night shift program. Among twenty-two private colleges, seven colleges were selected by using the lottery method and all government colleges were included. The total sample size was proportionally allocated to each of the colleges based on the number of night shift female students. From selected college students who are 2nd year and above was considered as the study population. Then finally, the study participants were selected by a systematic sampling technique using frame provided by the college registrar **N** (night shift female student in each college), n (proportionally allocated sample size to each college). K = N/n = 5.5 $\approx$ 5.

### Operational definition

#### Sexual violence

When a female student experienced at least one of the following: rape, attempted rape, and sexual harassment.

#### Rape

Is the act of forcing a female student through violent threats and deception to engage in sexual behaviors with penetration of the vagina [17].

#### Attempted rape

Efforts to rape someone which does not result in penetration with penetration of the vagina [18].

#### Sexual harassment

When a female student experienced one of the following: Is unwanted sexual behavior such as physical contacts or verbal comments, jokes, questions, kissing, hugging, and suggestions [18].

#### Substance use

Use of at least one of the following substances; alcohol, chat, Shisha, Hashish or drugs that are assumed to affect the level of thinking and increase the risk of involving in sexual violence [12].

#### Early sexual debut

When a female student experienced sexual activity at or before 18 years old.

### Data collection tools and procedure

The questionnaire adapted from the WHO multi-country questionnaire on violence against women and another internationally developed questionnaire on sexuality was used [19]. The questionnaire was made to measure socio-demographic, socio-economic characteristics and factors associated with sexual violence. Data was collected by nine diploma registrar workers and one BSc holder supervisor was assigned to control the data collection and assist data collectors. Before data collection was started, important communications were made with relevant institutions by using a letter prepared by the Hawassa university department of midwifery with different college’s heads. List of night shift female students were taken from the registrar of each college. The selected students were communicated and the purpose of the study was explained by the data facilitators. The questionnaire was distributed to every participant and collected on the same day to ensure confidentiality and prevent information contamination. The data was checked for the accuracy, completeness, and consistency of the information.

### Data analysis

The data were entered with Epi data version 3.1statistical software and analyzed by SPSS V.22. Bi variant analysis was carried out to see the association of each independent variable on the dependent variables. Then, Variables with a *p* value less than 0.25 in bi-variable logistic regression were entered to multivariate logistic regression to adjust the effect of confounders on the outcome variables. Finally, multiple logistic regression analysis techniques were carried out the statistical association was evaluated using odds ratio at 95% confidence interval and a *p* value less than 0.05. The Hosmer and Lemeshow (HL) test was used to check model fitness in our study. The HL test indicated that our final model was fit (0.906).

### Data quality assurance

The questionnaire prepared in English was translated into Amharic, and then back-translated into English to ensure consistency. The questionnaires were pretested on 5% [18] night shift students of the actual sample size in Golden star College in Hawassa city before the actual data collection period. Sensitive questions were tried to be placed later to minimize non-response rate and offensive reaction. The Training was given for both data collectors and supervisor. Data clean up and cross-checking was done before analysis.

## Result

### Socio-demographic characteristics

A total of 330 night shift female students were involved in the study making a response rate of 95.6%. The mean age of the participants was 24.96 years ± SD 6.01 years and the majority of the study participants 110 (33.3%) were in the age group of 20–24 years. 150 (45.5%) of them were protestant and 188 (57%) of the participants reported that they grew up in urban areas. Regarding respondent’s parents 93 (28.2%) of the father and 161 (48.8%) of the mother were with no formal education. 202 (61.2%) of the parents were living together, 32 (9.7%) were separated, 68 (20.6%) either father or mother alive and 28 (8.5%) both were not alive. The mean family monthly income of respondents was 2870.2 SD ± 3157 with a range of 300–13,500 birr. 151 (45.7%) of the student’s families had a monthly incomes greater than 3995 birr (Table 1).

**Table 1:** Socio-demographic characteristics of study participants in Hawassa city Sidama Region Southern Ethiopia, Nov 2020 (n = 330)

Characteristics	Frequency	Percent (%)
*Age grouped*		
15–19	69	20.9
20–24	110	33.3
25–29	84	25.5
≥ 30	67	20.3
*Religion*		
Protestant	150	45.5
Orthodox	115	34.8
Muslim	40	12.1
Catholic	25	7.6
*Marital status*		
Never Married	89	27
Single	26	7.9
Married	215	65.2
*Educational status*		
Certificate	80	24.2
Diploma	149	45.2
Degree	101	30.6
*Currently living with*		
Married	91	27.6
with parents	122	37.0
Relative	45	13.6
Lonely	43	13.0
Friends	29	8.8
*Childhood residence*		
Rural	142	43.0
Urban	188	57.0
*Having occupation*		
Yes	166	50.3
No	164	49.7
*Occupation (n* = *166)*		
Governmental	51	30.7
Self- employed	57	34.3
Labor	11	6.6
Merchant	35	21.1
House labor	12	7.2
*Monthly income*		
≤ 1025 birr	164	49.7
1026–3995 birr	61	18.5
> 3995 birr	105	31.8
*Lived for*		
Less than 7 year	134	40.6
Greater than 8 year	196	59.4
*Disability*		
Yes	50	15.2
No	280	84.8
*Father education level*		
No formal education	93	28.2
Primary	87	26.4
Secondary	71	21.5
more than secondary	79	23.9
*Mother education level*		
No formal education	161	48.8
Primary	83	25.2
Secondary	41	12.4
more than secondary	45	13.6
*Parental condition*		
Live together	202	61.2
Separated	32	9.7
Only the mother/father alive	68	20.6
Both of them not alive	28	8.5
*Monthly income*		
≤ 1025 birr	120	36.4
1026–3995 birr	59	17.9
> 3995 birr	151	45.7

### Substance use of students

About 89 (27%) of the participants stated that they have a history of alcohol drinking, of those who have a history of alcohol drinking, almost 80 (89.8%) drink occasionally. 56 (17%) of the students have a habit of chewing khat (Catha edulis). Only a few students, 16 (4.8%) reported having smoked cigarettes sometimes. 17 (5.2%) used substances like hashish 6 (1.8%) used cocaine at some point in their lifetime (Table 2).

**Table 2:** substance use among night shift female students in Hawassa city Sidama Region Southern Ethiopia, Nov 2020 (n = 330)

Characteristics	Frequency	Percent (%)
Alcohol		
Yes	89	27.0
No	241	73.0
Alcohol drinking frequency (n = 89)		
Always	9	10.1
Sometimes	80	89.9
How much you drinks (n = 89)		
1–3	61	68.5
≥ 4	28	31.5
Khat		
Yes	56	17.0
No	274	83.0
Khat chewing frequency (n = 56)		
Always	5	8.9
Sometimes	51	91.1
Cigarette		
Yes	16	4.8
No	314	95.2
Cigarette smoking frequency (n = 16)		
Always	1	6.25
Sometimes	15	93.75
Cocaine		
Yes	6	1.8
No	324	98.2
Hashes		
Yes	17	5.2
No	313	94.8

### Sexual history

From the total participants, 233 (70.6%) reported that they had sexual experience. 151 (64.8%) were started sexual intercourse at the age group of 15–18 years old. The mean age and SD for having the first sexual intercourse was found to be 12.5 ± 8.3 years. Among the study subjects, 83 (25.2%) of them had experienced two or more sexual partners in their lifetime. The major reasons to do sexual intercourse for the first time were 76 (32.6%) with in marriage (Table 3).

**Table 3:** sexual histories by respondents in Hawassa city Sidama Region Southern Ethiopia, Nov 2020 (n = 330)

Characteristics	Frequency	Percent (%)
*Started sexual intercourse*		
Yes	233	70.6
No	97	29.4
*Age at first sexual intercourse (n* = *233)*		
10–14 years	12	5.1
15–19 years	151	64.8
≥ 20	70	30.1
*Reason to do sexual intercourse*		
In marriage	76	32.6
Personal desire	25	10.7
Peer pressure	20	8.5
Partner false promise	20	8.5
Get money	14	6
Pass exam	12	5.1
Forced	66	28.3
*How many sexual partners do you have*		
Nothing	103	31.2
One	144	43.6
Two and more	83	25.2

### The prevalence of sexual violence

Out of the total study subjects, the prevalence of last 12 month sexual violence was 202 (61.2%) 95% CI (55.8, 66.4) which includes rape, attempted rape and sexual harassment. Last 12 months rape, attempted rape and sexual harassment was 46 (13.9%) 95% CI (10.6, 17.9), 23 (6.95) 95% CI (3.6, 10.9) and 163 (49.4%) respectively.

The lifetime rape was 92 (27.9%) 95% CI (23.3, 32.7) and among the raped victims, 49 (53.3%) have reported having at least one incident of rape in their lifetime. The lifetime attempted rape was 122 (37%) 95% CI (32.1, 42.4).from those 44 (13.3%) of the respondents have escaped from attempted forced sex by getting help from other people. At least once form of sexual harassment was reported by 276 (83.6%) in their lifetime. Most of those who reported having been harassed at least ones reported to face unwanted kiss by 121 (43.8%) in their lifetime and 84 (50.6%) face verbal jokes using advanced words and comment on physical appearance concerning sexuality in the last 12 months (Table 4).

**Table 4:** Magnitude of various forms of sexual violence among night shift female students In Hawassa town, Sidama region, Ethiopia Sep 2020 (n = 330)

Characteristics	Frequency	Percent (%)
*Lifetime prevalence of rape*		
Yes	92	27.9
No	238	72.1
*12-month prevalence of rape*		
Yes	46	13.9
No	284	86.1
*Lifetime prevalence attempt rape*		
Yes	122	37
No	208	63
*12-month prevalence of attempt rape*		
Yes	23	6.95
No	307	93.05
*How many time forced sex without consent*		
Once	49	53.3
Twice	33	35.9
Three times	7	7.6
Four and more	3	3.3
*Lifetime sexual harassment*		
Yes	276	83.6
No	54	16.4
*Past-12 month sexual harassment*		
Yes	163	49.4
No	167	50.6

### Perpetrators of rape

The victims were asked about their perpetuator and 22 (23.8%) of them reported that they were raped by boyfriends, followed by 18 (19.6%) by teachers. The mechanisms used to force the victims into sex differs were, 37 (40.2%) reported that it was after they were threatened of harm, following 26 (28.3%) by drinking a lot. The majority of the victims 37 (40.3%) reported that the age of perpetrators was > 35 years. Out of the total victims, 33 (35.9%) of them faced the rape in their home, following 25 (27.2%) of them in hotels (Table 5).

**Table 5:** Perpetuator and mechanism used to force sex among night shift female in Hawassa city Sidama Region Southern Ethiopia, Nov 2020 (n = 92)

Characteristics	Frequency	Percent (%)
*Perpetuator*		
Parent	16	17.4
Teacher	18	19.6
Student	2	2.2
Boy friend	22	23.8
Neighbor	17	18.5
Unknown	11	12.0
By group	6	6.5
*Mechanism perpetuator use*		
Beating	15	16.3
Pointed knife	7	7.6
Pointed gun	2	2.2
Threats harm	37	40.2
Forced drinking	26	28.3
Use drug	5	5.4
*Age of the perpetrator*		
15–24	29	31.4
25–34	26	28.3
> 35	37	40.3
*Place*		
My home	33	35.9
College	7	7.6
Jungle	10	10.9
Hotel	25	27.2
Friend home	14	15.2
Village	3	3.3

### Reporting about sexual violence

Two hundred sixty five (80.3%) of the study subjects perceived that binge night shift female student was a risk for sexual violence. 61 (66.3%) of the raped study subjects reported that they had told no one about their victimization. Only 15 (16.3%) of the rape was reported to the legal body. The victims of rape were asked about why they did not report to any body and gave different reasons. 30 (39%) were didn’t know what to do. 22 (23.9%) of them goes to the health facility and get medical care. Concerning the action taken against perpetrators, in the majority of the cases 72 (77.2%) no legal action was taken (Table 6).

**Table 6:** Reports the situation to the legal body among night shift female students in Hawassa city Sidama Region Southern Ethiopia, Nov 2020

Characteristics	Frequency	Percent (%)
*Bing night shift student risk for forced sex (n* = *330)*		
Yes	265	80.3
No	65	19.7
*For whom do u tell (n* = *92)*		
Nobody	61	66.3
Friend	22	23.9
Sister or brother	6	6.5
Parents	3	3.3
*Do you go to the health facility and getting help*		
Yes	22	23.9
No	70	76.1
*Report to a legal body*		
Yes	15	16.3
No	77	83.7
*For whom do u tell (n* = *15)*		
Kebele	2	13.3
Police	10	66.6
Elders	1	6.8
Women affairs	2	13.3
*Why you keep silent (n* = *77)*		
No know what to do	30	39
Afraid of parents	11	14.2
Afraid of public reaction	21	27.2
Afraid of perpetuator	12	15.8
No helpful legal body	3	3.8
*Action taken to perpetrator (n* = *92)*		
Nothing to taken	72	78.2
Imprisoned	13	14.1
Financial penalty	2	2.2
Financial compensation levied by elderly	3	3.3
Forced marriage	2	2.2

### Factors associated with sexual violence

On binary logistic regression analysis, father educational status, educational status of participants marital status, parent living condition, family income, work, being night shift, alcohol use, Impact of poor grade, and having sexual partners were associated with last 12 month sexual violence. But when adjusted only having father with no formal education (AOR = 2.39, 95% CI 1.04–5.33), drinking alcohol (AOR = 3.55, 95% CI 1.84–6.85), having sexual partners (AOR = 1.89, 95% CI 1.03, 3.5) and multiple sexual partners (AOR = 3.44, 95% CI 1.64, 7.2) were significantly associated with last 12 month sexual violence (Table 7).

**Table 7:** Bivariable and multivariate logistic regression analysis of factors associated with last 12 months Sexual Violence among night shift female college students in Hawassa city Sidama Region Southern Ethiopia, 2020

Variables	Last 12 month sexual violence (n = 330)		COR (95% CI)	AOR (95% CI)
	No (%)	Yes (%)		
*Father educational status*				
More than secondary	44 (55.7%)	35 (44.3%)	1.00	1.00
Illiterate	27 (29%)	66 (71%)	**3.07 (1.63–5.77)**	2.39 (1.04–5.33)*
Primary	30 (34.5%)	57 (65.5%)	2.38 (1.27–4.46)	1.76 (0.83–3.74)
Secondary	27 (38%)	44 (62%)	2.04 (1.06–3.93)	1.76 (0.80–3.85)
*Educational status of participants*				
Degree	28 (27.7%)	73 (72.3%)	1.00	1.00
Certificate	39 (48.8%)	41 (51.2%)	0.40 (0.21–0.74)	0.67 (0.32–1.4)
Diploma	61 (40.9%)	88 (59.1%)	0.55 (0.32–0.95)	0.70 (0.38–1.3)
*Marital status*				
Never married	25 (28.1%)	64 (71.9%)	1.00	1.00
Married	97 (45.1%)	118 (54.9%)	0.47 (0.27–0.81)	0.92 (0.48–1.79)
Single	6 (23.1%)	20 (76.9%)	1.30 (0.46–3.62)	1.3 (0.44–4.00)
*Parents living*				
Together	91 (45%)	111 (55%)	1.00	1.00
Separated	32 (26.5%)	68 (8%)	1.74 (1.05–2.88)	1.4 (0.78–2.5)
Both dead	5 (17.9%)	23 (82.1%)	3.77 (1.37–10.3)	2.3 (0.75–6.89)
*family income*				
> 3995 birr	64 (42.4%)	87 (57.6%)	1.00	1.00
≤ 1025 birr	39 (32.5%)	81 (67.5%)	1.52 (0.92–2.51)	1.05 (0.55–2.02)
1026–3995 birr	25 (42.4%)	34 (57.6%)	1.1 (0.54–1.83)	0.74 (0.35–1.6)
*Alcohol*				
No	113 (46.9%)	128 (53.1%)	1.00	1.00
Yes	15 (16.9%)	74 (83.1%)	**4.35 (2.36–8.01)**	3.55 (1.84–6.85)**
*Assume being night shift risky for violence*				
No	80 (44.9%)	98 (55.1%)	1.00	1.00
Yes	48 (31.6%)	104 (68.4%)	0.56 (0.36–0.88)	0.69 (0.40–1.17)
*Having work*				
Yes	51 (30.7%)	115 (69.3%)	1.00	1.00
No	77 (47%)	87 (53%)	0.50 (0.31–0.78)	1.3 (0.76–2.2)
*Impact of poor grade*				
No	92 (44.7%)	114 (55.3%)	1.00	1.00
Yes	36 (29%)	88 (59.9%)	0.50 (0.31–0.81)	0.7 (0.44–1.3)
*Sexual partner*				
None	62 (60.2%)	41 (39.8%)	1.00	**1.00**
One	48 (33.3%)	96 (66.7%)	**3.02 (1.78–5.11)**	1.89 (1.03–3.5)***
Two and more	18 (21.7%)	65 (78.3%)	**5.46 (2.83–10.5)**	3.44 (1.64–7.2)****

Bold indiactes *P* –value = *0.039, **<0.001, ***0.041 , ****0.001

## Discussion

Among a sample of 330 female students who participated in the study, the majority (70.6%) were sexually active. Of those who initiated sexual activity, 28.3% started sexual activity forcefully or as a result of rape. In this study, the prevalence of sexual violence and its associated factors were identified.

The findings from this study showed that more than half of students had experienced different forms of sexual violence for the last 12 months period. The prevalence of last 12 month sexual violence was 202 (61.2%) 95% CI (55.8, 66.4) which includes rape, attempted rape and sexual harassment. The result of this study was lower compared to study conducted among night shift secondary school and high school female students in Hawassa town indicated that prevalence of last 12 month sexual violence was 68.4% [4]. The difference might be due to that study used large sample size and study period.

The current study showed that the last 12 month prevalence of sexual violence among night shift female students is higher than a study conducted in Germany (5.4%), chili (9%) and Nigeria (11.8%) [20–22]. This variation could be due to a difference in culture, life style, socio-economy, and interventions made across the countries.

The finding also revealed that higher last 12 month prevalence of sexual violence than a country side study done in Ambo University (43.7%) [18], college female students in Nekemte town [12] and female high school students in Wolayta Sodo, (24.4%) [23]. This discrepancy might be due to the difference in the target population, study setting; student who lived in university is secured than a student who attends class from their home, operational definition of the problem. In this study, for example, any action against the women was considered as sexual violence and the nature of class time, because night time is more convenient for violent people, in this study.

The odds of having sexual violence in the last 12 months were 2.36 times higher in students whose father were no formal education than students with higher level educated. This is congruent to the study from Ambo which indicate that educated father have had protective effect to sexual violence of daughters than uneducated fathers [12]. This might be also the fact that educated fathers better informed decisions in the sexual sphere with their daughter than uneducated ones.

Finding of the study also depicts that students who ever experienced multiple sexual partners had 3.44 times more likely to have experienced sexual violence within the last 12 months compared to those students who haven’t had sexual partner. This finding is supported by a study conducted among female private college students in Bahirdar city [11]. And the study was done among female high school students in Soddo town [24]. This might be explained by the number of partners increases the chance of being sexually violated might be increased. Also, it could be explained by engaging in health–risk behaviors increase women’s vulnerability to forced sexual intercourse and the chance of getting abusive partners and peers among females with multiples sexual partners is by far higher than the referent groups.

Compared to those who have had no partners, those have sexual partner were 1.89 times more likely to experience sexual violence in the last 12 months. This is in line with a study conducted among night junior secondary school and high school female students in Hawassa town [4, 25]. This finding shows sexual violence is common among those who have an intimate relationship, which signifies less responsible sexual decision- making among partners.

The study indicated that those students who have had history of alcohol consumption were 3.54 times higher risk for sexual violence than their counterparts. This finding is similar to a study conducted among female students of Wolayta Sodo University [26] and among college female students in Nekemte [12] This might be due to alcohol consumption may predispose female to sexual violence because alcohol use cause loss of judgment, self-restrain in sexual intercourse and protective power of female from sexual violence.

The finding of this study has great public health significance in the journey of reducing the burden of sexual violence on college students, so the result of this finding helps responsible bodies, researchers, and health professionals to emphasize significant variables associated with sexual violence among women during their lifetime and research area.

## Conclusion

This study revealed that the last 12 month prevalence of sexual violence is high among night shift female college students in Hawassa city. The factors associated with sexual violence in this study were having illiterate father, drinking alcohol, Having sexual partner, and multiple sexual partners. As a result, interventions focusing on those identified factors by the concerned bodies could probably reduce the burden and consequences of induced asexual violence. Their fore, students should prevent themselves from health risky behaviors and Hawassa educational bureau should be work on awareness creation on women empowerment with in marriage and Further broad and longitudinal studies are needed to determine the predictors of the problem among female students at Hawassa and Ethiopia as a whole.

## Data Availability

The raw data documents are available upon request from the corresponding author.

## Abbreviations

AOR: Adjusted odd ratio
CI: Confidence interval
GBV: Gender-based violence
SPSS: Statistical package for social sciences
TVET: Technical vocational education and training
UN: United Nation
WHO: World Health Organization
